# Molecular Mechanism of Cold Tolerance of Centipedegrass Based on the Transcriptome

**DOI:** 10.3390/ijms24021265

**Published:** 2023-01-09

**Authors:** Yingjie Liu, Yi Xiong, Junming Zhao, Shiqie Bai, Daxu Li, Limin Chen, Junjie Feng, Yingzhu Li, Xiao Ma, Jianbo Zhang

**Affiliations:** 1 College of Grassland Science and Technology, Sichuan Agricultural University, Chengdu 611130, China; 2 Sichuan Academy of Grassland Science, Chengdu 610097, China

**Keywords:** centipedegrass, low-temperature stress, RNA-seq, transcription factor

## Abstract

Low temperature is an important limiting factor in the environment that affects the distribution, growth and development of warm-season grasses. Transcriptome sequencing has been widely used to mine candidate genes under low-temperature stress and other abiotic stresses. However, the molecular mechanism of centipedegrass in response to low-temperature stress was rarely reported. To understand the molecular mechanism of centipedegrass in response to low-temperature stress, we measured physiological indicators and sequenced the transcriptome of centipedegrass under different stress durations. Under cold stress, the SS content and APX activity of centipedegrass increased while the SOD activity decreased; the CAT activity, POD activity and flavonoid content first increased and then decreased; and the GSH-Px activity first decreased and then increased. Using full-length transcriptome and second-generation sequencing, we obtained 38.76 G subreads. These reads were integrated into 177,178 isoforms, and 885 differentially expressed transcripts were obtained. The expression of AUX_IAA and WRKY transcription factors and HSF transcription-influencing factors increased during cold stress. Through KEGG enrichment analysis, we determined that arginine and proline metabolism, plant circadian rhythm, plant hormone signal transduction and the flavonoid biosynthesis pathways played important roles in the cold stress resistance of centipedegrass. In addition, by using weighted gene coexpression network analysis (WGCNA), we determined that the turquoise module was significantly correlated with SS content and APX activity, while the blue module was significantly negatively correlated with POD and CAT activity. This paper is the first to report the response of centipedegrass to cold stress at the transcriptome level. Our results help to clarify the molecular mechanisms underlying the cold tolerance of warm-season grasses.

## 1. Introduction

*Eremochloa ophiuroides* (Munro) Hack, also known as Chinese lawn grass and centipedegrass, is an excellent warm-season lawn grass species that originated from China. It originated from the southern Yangtze River basin and is widely distributed in the southern and eastern parts of the United States, Southeast Asia, and the northern and eastern tropical regions of Australia [1]. The grass is famous for its tolerance of barren land, few pests and diseases, and low management intensity [2]. However, its poor cold resistance and short green period make it prone to withering and yellowing to different degrees in winter, which seriously affects its application scope, ornamental value on lawns and landscape effects, and has become the main limiting factor for its utilization. Therefore, understanding the cold resistance mechanism of centipedegrass and selecting new cold-resistant varieties are of great significance for its further popularization and utilization.

Low-temperature stress has a very important impact on warm-season grasses [3]. It can lead to plasma membrane peroxidation through reactive oxygen species (ROS), thus damaging the internal structure of cells [4]. Low-temperature stress can also directly inhibit the activity of chlorophyll-synthesis-related enzymes, thus affecting plant photosynthesis [5]. At the same time, it can have an indirect impact on photosynthesis by affecting other physiological processes in plants, such as causing water stress and blocking the transportation of photosynthetic products. After cold stress, the metabolic balance of plants is broken. To resist the damage caused by stress, plants can induce the synthesis of stress-related metabolites, such as ascorbic acid (Asa) and γ-aminobutyric acid [6], through signal transduction systems to repair the damage or achieve a new metabolic balance. Under low-temperature stress, the concentrations of amino acids, organic acids, sugars, sugar alcohols and other metabolites in bermudagrass significantly increase [7].

Regarding the cold stress response mechanism, studies on plant cold adaptation mechanisms have mainly focused on cold-induced gene expression and regulation processes, such as physiological regulation, hormone regulation and transcription factor regulation. The key response pathways also need to be activated through the molecular network and participate in the expression and signal transduction of specific stress response factors, thus developing into cold tolerance [8]. Many studies pointed out that the CBF signaling pathway can enhance the cold tolerance of bermudagrass [9], wheat [10], maize [11] and *Zoysia japonica* Steud [12]. In addition, hormone signal transduction plays a central regulatory role in the plant cold stress response and controls plant CBF-dependent and CBF-independent pathways. Abscisic acid (ABA) is an important abiotic stress-regulating hormone [13] that is mainly involved in abiotic stress signal transduction. Recent studies showed that ABA affects *COR* gene expression by regulating CBF transcription, thereby affecting the plant response to low temperature or cold stress. Some functional genes (such as transcription factors) can regulate the expression of cold resistance genes by participating in signal transduction and transcription. At present, many members of transcription factor families (*MYB*, *AP2/ERF*, *ICE* and *WRKY*) have been found and identified, and they are used as important regulators in the plant low-temperature response [14], thus effectively improving plant low-temperature tolerance.

An increasing number of studies were conducted on the transcriptome through the combination of PacBio and Illumina sequencing [15], which can not only take advantage of PacBio’s long reads to obtain a large number of complete full-length transcripts but also use Illumina’s high accuracy to calibrate PacBio data to obtain more reliable sequencing results. Transcriptome sequencing was widely used to study the cold resistance of warm-season turfgrasses, such as *Zoysia* [16] and *Paspalum notatum* [17]. However, a molecular mechanism for centipedegrass cold resistance was not previously discovered. Our study utilized both PacBio and Illumina sequencing strategies to investigate the molecular mechanism of centipedegrass under low-temperature conditions. Through the enrichment analysis of differentially expressed genes and WGCNA, we obtained the key genes and pathways involved in the cold resistance of centipedegrass. This study provides a theoretical basis for the study of the cold resistance of centipedegrass and other warm-season grasses.

## 2. Results

### 2.1. Physiological Changes in Centipedegrass under Low-Temperature Stress

**Figure 1:**
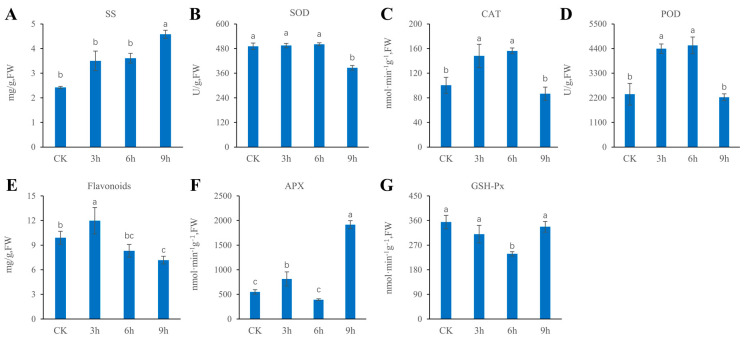
Effects of cold stress duration (CK, 3 h, 6 h and 9 h) on physiological indices of centipedegrass. (**A**) Soluble sugar content, (**B**) SOD activity, (**C**) CAT activity, (**D**) POD activity, (**E**) flavonoid content, (**F**) APX activity and (**G**) GSH-Px activity. Data are given as the mean ± SD (different letters indicate significant differences at *p* < 0.05 via Duncan’s test).

Under low-temperature stress, the content of soluble sugar (SS) in the leaves of centipedegrass gradually increased, among which the SS content at 9 h was approximately 200% higher than that of the control (CK) (Figure 1A). In addition, under low-temperature treatment, the activity of superoxide dismutase (SOD) was significantly decreased at 9 h (*p* < 0.05, Figure 1B). The activities of catalase (CAT) and peroxidase (POD) and the flavonoid content showed a tendency to first increase and then decrease (Figure 1C–E). The low-temperature treatment also significantly affected the ascorbic acid peroxidase (APX) activity of centipedegrass, and the APX activity reached a maximum value of 1913 nmol/min/g at 9 h (Figure 1F). The activity of glutathione peroxidase (GSH Px) first decreased and then increased, among which the GSH Px activity at 6 h was significantly lower than that of the CK (*p* < 0.05, Figure 1G).

### 2.2. RNA-Seq Results

The full-length transcriptome of centipedegrass under cold stress was sequenced based on cDNA libraries constructed from the CK and treatments. After data cleaning, a total of 16,244,578 (38.76 G) subreads were obtained. By filtering subreads based on full passes ≥1, we obtained 331,513 circular consensus sequences (CCSs). Moreover, 281,066 full-length nonchimeric (FLNC) reads were detected with an average length of 2652 bp. The FLNC reads were hierarchically clustered to obtain consensus sequences. Then, after correcting the consensus sequences using the Quiver algorithm, 172,733 high-quality sequences and 4127 low-quality sequences were obtained. To improve the sequence accuracy, the low-quality consensus sequence was corrected with the corresponding Illumina RNA-seq data using proovread software [18]. Using CD-HIT, redundant sequences were removed from the transcripts [19]. A total of 127,142 nonredundant transcript sequences were ultimately acquired and used for further analysis (Table 1).

Centipedegrass cDNA libraries created with samples from the CK and three cold stress treatments (a total of twelve samples) were sequenced using the Illumina RNA-seq strategy. A total of 84,519,789,530 bp of clean data and 282,243,962 clean reads were obtained. In addition, the average Q30 reached 94.33% and the average GC content reached 50.78% (Table S1).

### 2.3. Functional Annotation of Isoforms

A total of 95.74% (121,738 of 127,142) of the isoforms were successfully annotated in the NR, KEGG, KOG, COG, GO, Pfam and SwissProt databases (Table S2). All isoforms of centipedegrass were aligned with the NR database, and the results showed that *Sorghum bicolor* possessed the highest similarity (25,799, 21.42%) with centipedegrass, followed by *Zea mays* (11,077, 9.20%) (Figure S1). A total of 92,875 (73.05%) isoforms were assigned to GO terms in the cellular component, molecular function and biological process categories; these isoforms were further classified into 50 GO terms (Figure S2). In the cellular component category, most of the isoforms were categorized into two subcategories: cell part (52,703) and cell (52,514). In the molecular function category, the number of isoforms involved in catalytic activity (49,701) and binding (48,877) was much greater than that for other subcategories. In the biological process category, the metabolic process, cellular process and single-organism process contained a vast number of isoforms (>30,000 each).

### 2.4. Long Noncoding RNA Prediction and Transcription Factor Identification

Through the alignment with seven databases, most of the full-length sequences in the centipedegrass transcriptome data were well annotated. Four databases, namely, CNCI, CPC, CPAT and Pfam, were then used to predict the coding ability of the isoforms, and the “noncoding” results predicted by these software programs were considered to be the final lncRNA results; that is, a total of 1785 lncRNAs were obtained (Figure S3).

Transcription factors (TFs) refer to proteins that can bind to specific nucleotide sequences upstream of a gene. These proteins can regulate the binding of RNA polymerase to DNA templates, thereby regulating gene transcription. In this study, a total of 12,165 sequences belonging to 5178 TF families were identified in 127,142 full-length transcript sequences using iTAK software [20] (Figure S4).

### 2.5. Differentially Expressed Genes (DEGs) in Response to Low-Temperature Stress

**Figure 2:**
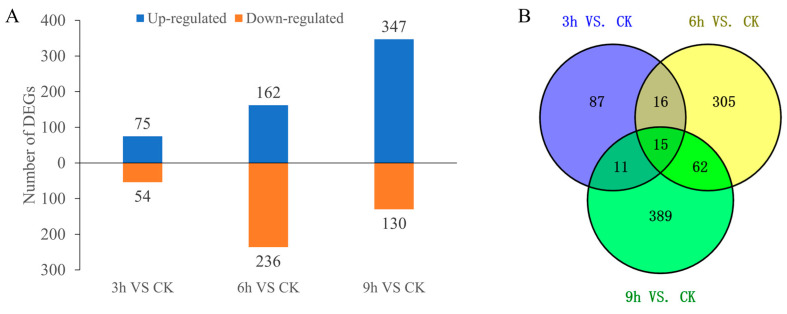
DEG statistics for centipedegrass under low-temperature stress compared with the CK. (**A**) Number of DEGs under different stress durations compared with the CK; (**B**) the DEGs at different time points compared with the CK are shown in a Venn diagram.

To understand how cold stress affects the gene expression of centipedegrass, gene expression was compared between time points (CK vs. 3 h, CK vs. 6 h and CK vs. 9 h). A total of 129 DEGs were identified between 3 h and the CK, among which 75 genes were upregulated and 54 were downregulated. In the case of 6 h and the CK, 398 DEGs were detected, with 162 upregulated genes and 236 downregulated genes. When comparing the 9 h and CK groups, 477 DEGs were identified, with 347 genes being upregulated and 130 genes being downregulated (Figure 2A). A total of 15 DEGs were shared between all comparisons (Figure 2B).

### 2.6. GO and KEGG Enrichment Analysis of DEGs

**Figure 3:**
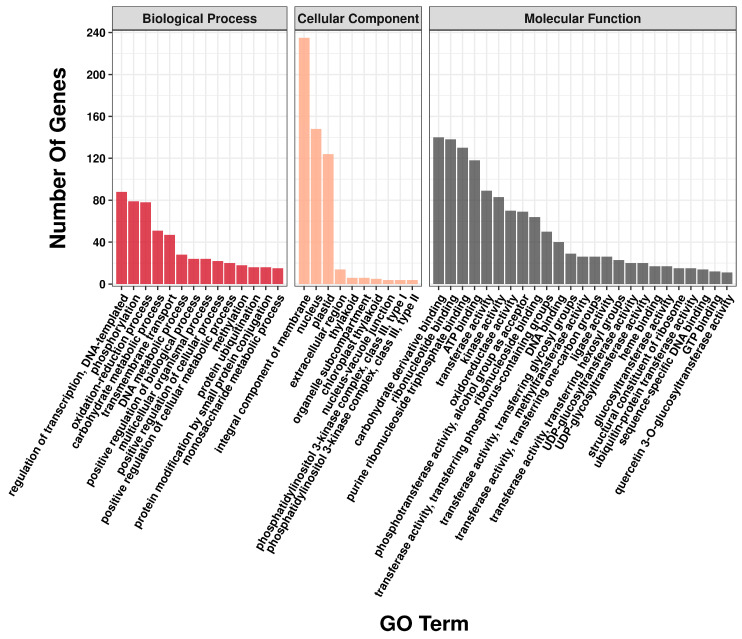
Analysis of 885 DEGs enriched for GO terms in centipedegrass.

The GO enrichment results showed that a total of 885 DEGs were classified into 243 GO terms. The DEGs were involved mostly in cellular components, accounting for 47.69% of the total DEGs. In terms of molecular function, the number of genes involved in carbohydrate derivative binding was the highest, with a total of 140. In biological processes, DEGs were mainly focused on the regulation of transcription, DNA-templated, phosphorylation and oxidation–reduction processes. Among all the GO terms, the integral component of the membrane in the cellular component was the most enriched, with a total of 235 DEGs (Figure 3).

An enrichment analysis of KEGG pathways was also conducted. In different treatments, the DEGs were mainly enriched in arginine and proline metabolism, plant circadian rhythm pathway, plant hormone signal transduction and flavonoid biosynthesis (Figure S5; Table S3). The plant circadian rhythm pathway was enriched at both 6 h and 9 h. All these pathways work together to form a set of signaling networks to cope with cold stress.

### 2.7. Arginine and Proline Metabolism

**Figure 4:**
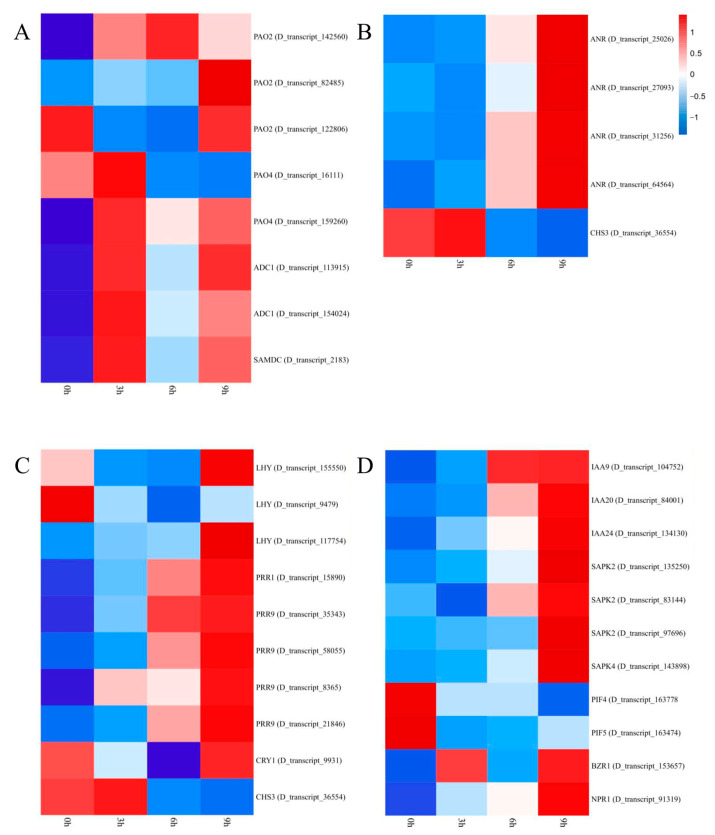
Heatmaps showing the expression of genes related to the arginine and proline metabolism pathways (**A**), flavonoid biosynthesis pathway (**B**), plant circadian rhythm pathway (**C**) and plant hormone signal transduction pathway (**D**).

A total of seven DEGs that participate in the metabolic pathway for arginine and proline were upregulated at 3 h, among which four genes were annotated as polyamine oxidase (POA) (*D_transcript_159260*, *D_transcript_142560*, *D_transcript_82485* and *D_transcript_16111*). Two arginine decarboxylase (ADC) DEGs (*D_transcript_113915* and *D_transcript_154024*) were significantly upregulated at 3 h. S-adenosylmethionine (SAMDC) plays an important role in arginine metabolism. In this experiment, one SAMDC gene (*D_transcript_2183*) showed a tendency to first increase and then decrease with the extension of cold stress. Considering that most of the DEGs were annotated in the arginine and proline pathways at the beginning of cold stress (Figure 4A), the contents of arginine and proline could be considered potential markers to deduce the degree of cold stress in centipedegrass and other warm-season grasses.

### 2.8. Flavonoid Biosynthesis

A lower temperature is conducive to the accumulation of flavonoids, and Ca^2+^/ABA transmits signals to downstream carbohydrate and flavonoid synthase genes to promote the accumulation of sugars, thus providing a material basis for flavonoids and increasing flavonoid metabolism enzyme activity. In this pathway, we identified five significant DEGs. One DEG annotated as chalcone synthase 3 (CHS3, *D_transscript_36554*) was upregulated at 3 hours and then significantly decreased, and four DEGs related to anthocyanin reductase were upregulated (*D_transcript_25026*, *D_transcript_27093* and *D_transcript_31256*) (Figure 4B).

### 2.9. Plant Circadian Rhythm Pathway

**Figure 5:**
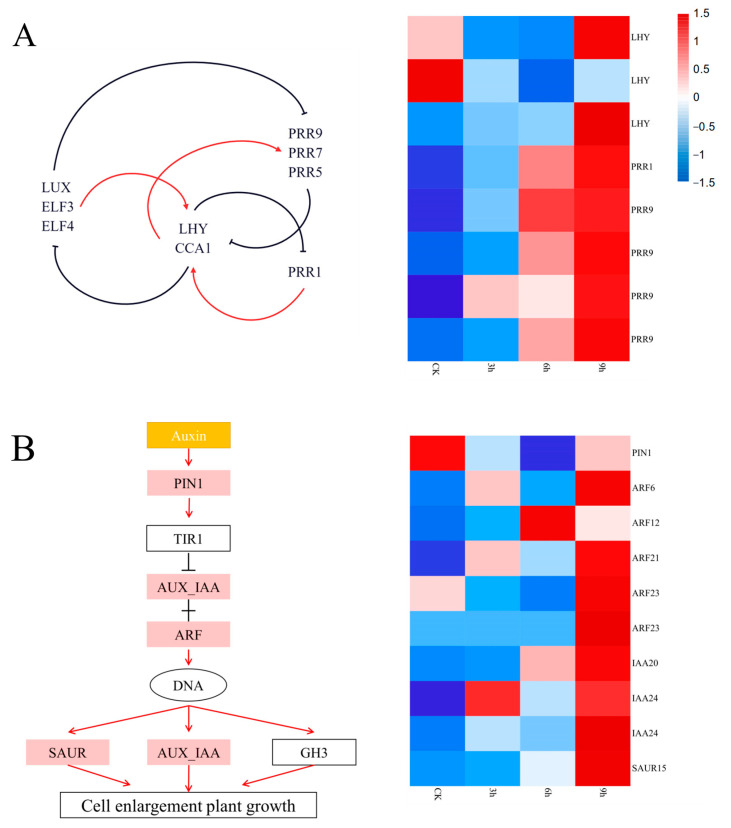
DEG involvement in different pathways in centipedegrass under low-temperature stress. (**A**) Central oscillator in the plant circadian rhythm pathway; (**B**) auxin polar transport and signal transduction pathway. The red arrow indicates activation and the black cutoff indicates inhibition.

The regulation of circadian rhythm plays an important role in plant adaptation to low temperatures. CBF (CRT/DRE binding factor) is an important signal transduction pathway for plants to respond to low-temperature stress. The upstream inducer of CBF expression (ICE) activates the expression of CBF, and then the downstream cold-regulated gene (COR) is activated by CBF to increase the cold resistance of plants. CBF expression is induced in the daytime but is hardly expressed at night. In this experiment, we identified three MYB-related transcription factors related to late elongated hypocotyl (LHY), in which two DEGs were first downregulated and then upregulated (*D_transcript_9479* and *D_transcript_155550*) and one DEG was upregulated (*D_transcript_117754*). In addition, we identified five DEGs (*D_transcript_35343*, *D_transcript_58055*, D_transcript_8365 and *D_transcript_21846*) related to pseudoresponse regulator 9 (PRR9) and pseudoresponse regulator 1 (PRR1), where their expression showed an upward trend. Cold stress stimulated the LHY response and promoted the expression of *PRR1* and *PRR9* (Figure 4C and Figure 5A).

### 2.10. Plant hormone Signal Transduction

Hormones regulate plant responses to low temperatures by modulating complex cascades. In the metabolic pathway of plant hormone signal transduction, we observed 11 DEGs, as indicated in Figure 4. Among them, three DEGs (*D_transcript_135250*, *D_transcript_143898* and *D_transcript_97696*) encoding serine/threonine protein kinase (SAPK) increased gradually and one DEG (*D_transcript_83144*) encoding SAPK was first downregulated and then significantly upregulated. All three AUX/IAA DEGs (*D_transcript_104752*, *D_transcript_134130* and *D_transcript_84001*) were upregulated under cold stress. In addition, we identified two upregulated DEGs (*D_transcript_153657* and *D_transcript_91319*) annotated with the ethylene signaling pathways and the salicylic acid pathway. Two downregulated DEGs (*D_transcript_163474* and *D_transcript_163778*) were related to phytochrome-interacting factor 4 (PIF4) (Figure 4D and Figure 5B).

### 2.11. Transcription Factors (TFs) and Transcriptional Regulation Factors (TRs)

**Figure 6:**
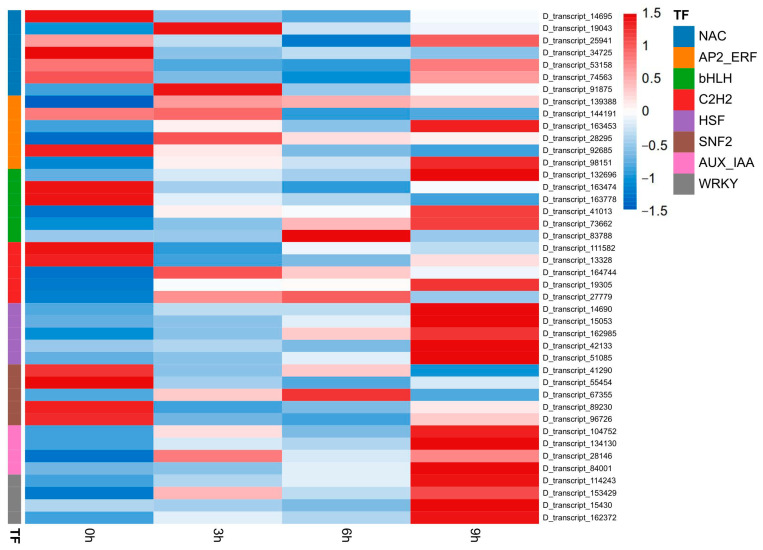
TF and TR expression profiles between centipedegrass samples under low-temperature stress based on FPKM values. Red and blue indicate high and low expression levels, respectively.

An essential role of transcription factors (TFs) in the response of plants to abiotic stresses was established. Further analysis of DEG regulation revealed 82 TFs regulating 885 DEGs, and these TFs were from 27 TF families, such as NAC, AP2_ERF and WRKY. We analyzed the expression patterns of 42 DEGs in the transcription factor families with greater than 4 DEGs. We drew a heatmap of the TFs with altered abundance to describe the transcript abundance profile of each TF family (Figure 6). Most DEGs of the NAC TF family were downregulated at 3 h, and the expressions of all DEGs of the HSF, AUX-IAA and WRKY transcription factor families were upregulated at 9 h.

### 2.12. Weighted Gene Coexpression Network Analysis (WGCNA)

**Figure 7:**
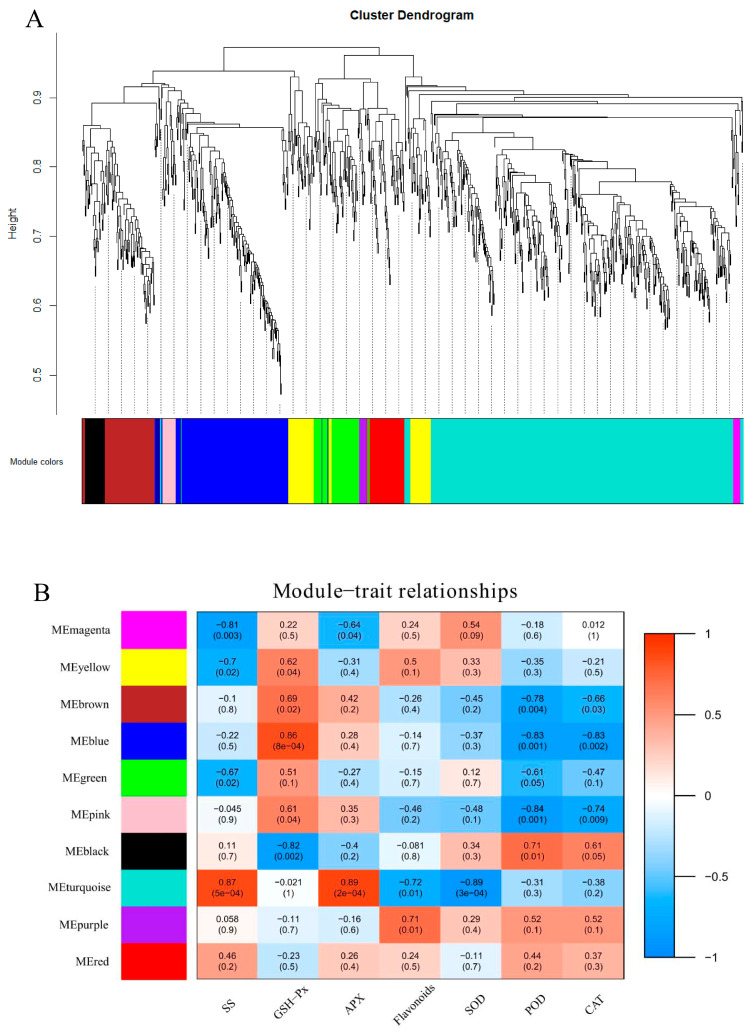
Identification and correlation analysis of WGCNA modules in centipedegrass under low-temperature stress. (**A**) Hierarchical clustering tree of 10 coexpression modules identified using WGCNA. (**B**) Correlation analysis of 10 modules and 7 physiological traits. Correlation coefficients are color-coded from (−1) (blue) to (1) (red) and the associated p-values are indicated in parentheses.

**Figure 8:**
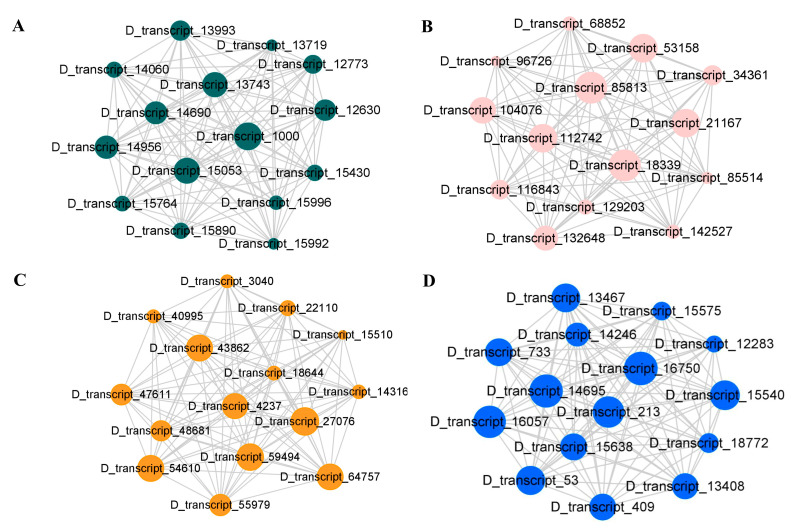
Hub genes in the candidate modules and their intramodular relationship. (**A**) Top 15 hub genes in turquoise, (**B**) top 15 hub genes in pink, (**C**) top 15 hub genes in black and (**D**) top 15 hub genes in blue.

To identify the genes responsible for cold stress resistance in centipedegrass, we conducted WGCNA using 885 DEGs and a total of 10 modules were identified (Figure 7A). SS and APX were positively correlated with the turquoise module, while POD was negatively correlated with it. The pink and blue modules were negatively correlated with GSH-Px but were positively correlated with POD (Figure 7B). A central hub gene in each network was defined as a gene whose connectivity with other genes within the same module was the highest; these genes might function as the principal regulator in these modules. We analyzed the hub genes of modules with correlations greater than 0.8, and the 15 genes with top connectivity were considered the central hub genes in each network (Figure 8). Our research identified some genes that were involved in the transmission and regulation of stress signals, including calcium-dependent protein kinase (CDPK) (*D_transcript_16057*), signal transduction mechanisms MA3 domain (*D_transcript_18339*), proline-rich receptor-like protein kinase (PERK10)(*D_transcript_59494*) and cysteine-rich receptor-like protein kinases (*D_transcript_15540*). Furthermore, several transcripts involved in the pentose phosphate pathway and glycolysis/gluconeogenesis, including glyceraldehyde-3-phosphate dehydrogenase A (GAPDH, *D_transcript_43862*), phosphoacetylglucosamine mutase (*D_transcript_21167*) and α-L-arabinofuranosidase 1 (*D_transcript_85813*), were identified.

### 2.13. qRT-PCR Validation of the DEGs

Eight genes were randomly selected for qRT–PCR analysis to validate the RNA-seq results. Based on these results, the qRT–PCR data were generally consistent with the RNA-seq data, which confirmed the reliability of the RNA-seq analyses in this study (Figure S6).

## 3. Discussion

China is one of the countries with the most abundant varieties of warm-season turfgrasses in the world, but low temperatures limit the promotion of warm-season turfgrasses in northern China. To cope with cold stress, warm-season turfgrass must adjust its morphological, physiological, biochemical and metabolic processes to survive [20]. To identify the key genes that contribute to centipedegrass cold resistance, we treated the grass with low-temperature stress and carried out transcriptome sequencing and physiological index determination. The transcriptome results showed that pathways involving amino acid transport and metabolism, hormone signal transduction, flavonoid biosynthesis and plant circadian rhythm played significant roles in the low-temperature stress response of centipedegrass.

### 3.1. Signal Transduction

Hormone signal transduction pathways were widely discussed among the major contributors to the plant cold response. Signal transduction in plant cells includes intracellular signal transduction, signal transmembrane conversion on the cell membrane, intercellular signal transmission and protein phosphorylation [20]. Under cold stress, the expression of *cyclic nucleotide-gated channel* (*CNGC17)* decreased, and the intracellular Ca^2+^ concentration increased [21]; then, the corresponding calcium signal was generated after the regulation of relevant transporters. Ca^2+^ signals are usually transduced by CDPKs, Ca M-like proteins (CMLs) and calcineurin B-like proteins (CBLs) [22]. Two DEGs (*D_transcript_152083* and *D_transcript_59016*) related to CML signals were found in the current study, both of which were upregulated. However, one DEG (*D_transcript_16057*) related to CDPK13 signaling was downregulated. In previous studies, CMLs were shown to interact with *WRKY* and other transcription factors to improve plant tolerance to abiotic stresses [23]. Research on rice showed that the *OsCDPK17* and *OsCDPK24* genes were significantly upregulated under low-temperature treatment, and overexpression of *OsCDPK17* and *OsCDPK24* could significantly improve the ability of rice to withstand low temperatures [24]. Our study also found two DEGs (*D_transcript_23456* and *D_transcript_82334*) that encoded CBLs, and their expression showed a trend of first decreasing and then increasing. In low-temperature signal transduction, CBLs interact with protein kinases (CIPKs) to transmit Ca^2+^ signals to the terminal, which could further regulate the expression of downstream *COR* genes and finally allow plants to adapt to low temperatures [25,26,27]. We speculated that these DEGs involved in Ca^2+^ signal transduction might play important roles in the response process to low-temperature stress.

The MAPK signal cascade is a common signal transduction module that can transform external stimuli into cell responses and participate in a variety of biological processes [28]. Many of these genes were identified in other plants [29]. Research showed that the MAPK cascade plays a positive role in plant cold tolerance [30]. Under low-temperature stress, *GhRaf19*-overexpressing tobacco possessed higher cold tolerance [31]. Our experiment identified that one DEG (*D_transcript_112755*) related to serine/threonine protein kinase was upregulated under cold stress. This also confirmed that MAPK-signaling-pathway-related genes might participate in the response to low-temperature stress. *SnRK* (*Sucrose nonferrous 1-related protein kinase*) plays an important role in regulating plant hormone signal transduction, stress response, and growth and development, as well as other physiological processes [32]. Many studies showed that *SnRK2* plays an important role in the stress response of gramineous plants [33]. In this study, all four differentially expressed *SnRK* genes (*D_transcript_135250, D_transcript_143898, D_transcript_83144* and *D_transcript_97696*) were upregulated. We speculate that the upregulated expression of the *SAPK2* gene contributes to the improvement of cold resistance in centipedegrass.

Plant growth and development are primarily regulated by indole-3-acetic acid (IAA) and other plant hormones. In published studies, exogenous IAA can alter the relative expression of the cold-related gene *ICE1* and reduce the damage caused by cold stress [34]. Auxin can activate *PIN1* expression and then regulate *TIR1* to remove *ARF* inhibition, thus initiating the expression of *AUX-IAA* and *SAUR* to increase the cold resistance of plants [35]. In our study, some genes related to *PIN1*, *ARF*, *AUX-IAA* and SAUR were differentially expressed under cold stress, which suggested that the plant hormones, especially IAA, participated in the cold stress response of centipedegrass (Figure 5B).

### 3.2. Arginine Metabolism

Arginine is an important nitrogen storage nutrient in plants and a precursor of the biosynthesis of polyamines (PAs) and nitric oxide (NO). PAs and NO, as signaling molecules, participate in many physiological processes in plants [36,37]. A study on tomato fruits showed that low-temperature stress significantly stimulated the activation of key enzymes of arginine catabolism, including *arginase*, *arginine decarboxylase* (*ADC*) and *nitric oxide synthase* (*NOS*) [38]. In our study, there were five DEGs (*D_transcript_159260, D_transcript_142560, D_transcript_82485, D_transcript_122806* and *D_transcript_16111*) related to polyamine oxidase, and most of them were upregulated. *S-adenosylmethionine decarboxylase* (*SAMDC*), as one of the three key enzymes *(ADC*, *ODC* and *SAMDC*) in polyamine synthesis, plays an important role in regulating endogenous polyamine content, anabolism, plant growth and development, and stress resistance [39]. We found that *SAMDC*-related DEGs (*D_transcript_2183*) were significantly upregulated at 3 h. In addition, two DEGs (*D_transcript_113915* and *D_transcript_154024*) related to *ADC1* were also significantly upregulated during stress. Research showed that putrescine could improve the ability of wheat to cope with abiotic stress, and overexpression of the *ADC1* gene could increase the content of putrescine in the seeds of wheat [40].

### 3.3. Circadian Rhythm in Plants

Plant circadian rhythm controls many important physiological processes of plants and endows plants with adaptability to the diurnal periodic fluctuations of external environmental factors, such as light and temperature, so that plants can better adapt to the environment when subjected to abiotic stress [41]. Studies on *Lolium perenne* and *Elymus dahuricus* also found that cold stress affects the circadian rhythm [42,43]. The central oscillator of circadian rhythm is composed of three proteins: circadian clock associated 1 (CCA1), pseudo response regulator (PRRs) and late elongated hypocotyl (LHY) [44]. CCA1 and LHY are homologous proteins and members of the *MYB* transcription factor family. Studies in *Arabidopsis* showed that LHY can regulate plant cold resistance by controlling the expression of the *CBF* gene [45]. In this study, there were three DEGs (*D_transcript_155550*, *D_transcript_9479* and *D_transcript_117754*) that encoded LHY, two of which were upregulated (*D_transcript_155550* and *D_transcript_117754*). In addition, we found one DEG (*D_transcript_15890*) related to *PRR1* and four DEGs related to *PRR5* (*D_transcript_35343*, *D_transcript_58055*, *D_transcript_8365* and *D_transcript_21846*), all of which were upregulated. It is speculated that the genes encoding PRRs play an important role in the survival environment of centipedegrass to adapt to periodic fluctuations in temperature (Figure 5A).

### 3.4. Flavonoid Biosynthesis

As antioxidants, flavonoids can eliminate and alleviate the damage caused by abiotic stresses, such as drought and high salt concentrations [46]. Under abiotic stresses, various physiological and biochemical changes occur in plant cells, resulting in the production of a large number of reactive oxygen free radicals and serious damage to cell structures. Flavonoids can also promote the expression of catalase, superoxide dismutase, glutathione peroxidase and other enzymatic antioxidants in cells [47]. Schulz et al. confirmed that flavonoids are among the determinants of cold acclimation and frost resistance in *Arabidopsis thaliana* [48]. Flavonoid synthesis in plants is strictly regulated by a complex network [49]. Anthocyanin reductase (ANR), as a key enzyme in flavonoid synthesis, plays an important role in regulating the content of flavonoids in plants and the changes in leaf color. All four DEGs (*D_transcript_25026*, *D_transcript_27093*, *D_transcript_31256* and *D_transcript_64564*) related to *ANR* identified in this study were upregulated.

### 3.5. Transcription Factors (TFs) and Transcriptional Regulation Factors (TRs)

Transcription factors and transcription regulation factors are important regulators of plants in response to abiotic stress. Many transcription factors, including WRKY, MYB and NAC, were confirmed to play an important regulatory role in salt stress [50], drought stress [51], low-temperature stress [52] and heavy metal stress [53]. In this study, 82 transcription factors and transcription regulation factors were annotated from 885 DEGs. WRKY is one of the largest transcription factor families in plants. All four WRKY family genes (WRKY2, WRKY31, WRKY34 and WRKY41) annotated in our study were upregulated at 9 h. The WRKY2 gene was shown to regulate plant circadian rhythm and improve the ability of plants to cope with low-temperature stress by promoting the expression of CCA1 and LHY [54]. The WRKY31 gene was also shown to improve plant cold tolerance in combination with the key promoter of ABA biosynthesis to promote ABA synthesis [55]. Similar to WRKY transcription factors, HSF transcription factors also play an important role in the abiotic stress responses of many plants. Our research annotated five HSF family genes, three of which were related to HSFA3 (D_transcript_14690, D_transcript_15053 and D_transcript_51085). In a study on *Arabidopsis*, HSFA3 positively regulated the synthesis of galactinol synthase and improved the resistance of plants to stress by scavenging free radicals [56]. Our study also verified this point, and all three DEGs that encoded HSFA3 were upregulated at 9 h.

## 4. Materials and Methods

### 4.1. Plant Materials and Low-Temperature Treatment

The *Eremochloa ophiuroides* cultivar “Wuling” used in this study was from the Sichuan Academy of Grassland Science (Chengdu, China). Ten stolons of centipedegrass were inserted into pots, cultured in an incubator for 90 days (28 °C, 8000 lux, 60% humidity and 14 h/d light) and then placed into a low-temperature artificial climate box for low-temperature stress. The temperature of the low-temperature artificial climate box was set to 4 °C, the light intensity was 8000 lux and the relative humidity was 60%. The leaves of the CK and cold-treated plants were collected at different time points (0, 3 h, 6 h and 9 h); each process was repeated three times. The collected samples were immediately frozen in liquid nitrogen for RNA extraction and the determination of physiological indicators (SS, CAT, POD, SOD, GSH-Px, APX and flavonoids). All experiments were repeated three times. Data were presented as the means and standard errors, and then ANOVA was used to analyze them for analysis. Multiple comparisons were performed via Duncan’s test (*p* < 0.05) using the software SPSS 20 software.

### 4.2. RNA Extraction, Library Construction and Sequencing

Using the TRIzol kit (Invitrogen, CA, USA) and its instructions, total RNA was extracted from 12 centipedegrass leaf samples. Each sample was electrophoresed on an agarose gel to determine its RNA quality. The RNA purity was determined using a NanoPhotometer^®^ spectrophotometer (IMPLEN, Munich, Germany). Using SMARTer™, the PCR cDNA Synthesis Kit (Pacific Biosciences, San Diego, CA, USA) synthesized the full-length cDNA of mRNA, amplified the full-length cDNA using PCR, repaired the ends of the full-length cDNA, connected the SMRT dumbbell-shaped connector and finally performed exonuclease digestion to obtain the cDNA library. The Pacific Biosciences DNA Template Prep Kit 2.0 was used in the construction of SMRTbell iso-seq libraries. Sequencing was performed on the RS II platform (Pacific Biosciences, San Diego, CA, USA). To construct the cDNA library for SGS, the manufacturer provided a protocol for using the NEBNext^®^ UltraTM RNA Library Prep Kit for Illumina^®^ (NEB, Beverly, MA, USA). SGS produced 125 bp paired-end sequence reads (2 × 125 bp) based on qualified libraries applied to Illumina’s NovaSeq 6000 (Pacific Biosciences, San Diego, CA, USA). The Biomarker Technology Co. (Beijing, China) performed the high-throughput sequencing (TGS and SGS) for this study.

### 4.3. De Novo Assembly

A circular consensus sequence (CCS) was generated from PacBio raw reads as the first step. The poly-A tail signal and the 5’ and 3’ cDNA primers in CCS were then used to determine full-length nonchimeric (FLNC) transcripts. By clustering full-length sequences from the same transcript, similar full-length sequences were grouped into clusters, and each cluster produced a consensus sequence. The consensus sequences were corrected to obtain high-quality sequences for subsequent analysis. CD-HIT (v4.6.8) was used to remove the redundancy from high-quality FL transcripts derived from the Iso-Seq method (identity > 0.99) [19].

### 4.4. Expression Calculation and Differential Expression Analysis

RSEM (v1.3.3) [57] software was used to quantify the expression level of the transcripts through the location information of mapped reads from the three generations of transcripts. To make the number of fragments truly reflect the transcript expression level, the read counts of each transcript were normalized to the transcript length and total mapped read counts within each sample and expressed as FPKM. An analysis of differential expression was performed using R DESeq (Version 1.18.0) [58]. The *p*-values were adjusted based on the Benjamini–Hochberg method [59].

### 4.5. Functional Annotation of Transcripts

Annotations were based on BLASTX hits in eight public databases containing nonredundant transcripts, including GO (Gene Ontology), KEGG (Kyoto Encyclopedia of Genes and Genomes), COG (Clusters of Orthologous Groups of proteins), Pfam (Protein family), KOG (Eukaryotic Ortholog Groups), egg-NOG (Evolutionary Genealogy of Genes: Nonsupervised Orthologous Groups), Swiss-Prot (A manually annotated and reviewed protein sequence database) and NR (NCBI nonredundant protein sequences). E-values were set to 10^−5^ for all searches. GO enrichment analysis was carried out using the Wallenius noncentral hypergeometric distribution with the GO seq R package [60]. KOBAS software (v3.0) [61,62] was used for the statistical analysis of KEGG pathway enrichment.

### 4.6. Quantitative Real-Time PCR (qRT–PCR) Analysis

A RevertAid cDNA Synthesis Kit (AiDLAB Biotech, Beijing, China) was used to synthesize cDNA for qPCR. Primers were designed with Beacon Designer (v7.9) and synthesized by Sangon Biotech (Shanghai China). An SYBR Green qPCR Mix (DF Biotech., ChengDu, China) and a qTOWER 2.0/2.2 Quantitative Real-Time PCR Thermal Cycler (Analytik Jena AG, Jena, Germany) were used to perform real-time PCR (qRT–PCR). For the quantification of gene expression, the FC = 2^−ΔΔCt^ method was used with U6 as an internal reference. Table S4 shows the sequences of all primers.

### 4.7. WGCNA Analyses

The R package was used to analyze gene expression networks using weighted gene coexpression networks (WGCNA) [63]. A 1-step network construction process and module detection were used to construct the network, as well as for the identification of each module based on its function. Eigenvalues were used to indicate the gene associations between modules and within physiological indices at different time points. Based on physiological indicators, all coexpressed DEGs were divided into 10 modules. Cytoscape (v.3.7.2) was used to visualize each network.

## 5. Conclusions

PacBio single-molecule long-read sequencing and Illumina RNA-seq data were combined to reveal the cold-stress response mechanisms of centipedegrass. A total of 885 DEGs were identified, and some members of the *AUX_IAA* and *WRKY* gene families were significantly upregulated at 9 h; thus, we inferred that 9 h is a key time point for centipedegrass to respond to cold stress. We found that several pathways, including signal transduction, the circadian rhythm in plants, and flavonoid biosynthesis, might play important roles in the cold stress response of centipedegrass. These transcriptome data could provide valuable resources for further studies on warm-season turfgrasses.

## Figures and Tables

**Table 1:** The characterization of the full-length transcriptome of centipedegrass.

Sample	TML
Subreads base (G)	38.76
Subreads number	16,244,578
Average subreads length	2386
CCS	331,513
FLNC	281,066
Average FLNC read length	2652
FLNC/CCS	0.84
Total nucleotides	349,159,670
Total number	127,142
Mean length	2746
Max length	13,081
N50	3089
N90	1773

TML represents mixed samples containing treated and control samples.

## Data Availability

Sequence data from the present study were submitted to the CNGB with accession code CNP0003518.

## Supplementary Materials

The supporting information can be downloaded from https://www.mdpi.com/article/10.3390/ijms24021265/s1.
