# Supplementary material for: Molecular Mechanism of Cold Tolerance of Centipedegrass Based on the Transcriptome
Source: Int J Mol Sci. 2023 Jan 9;24(2):1265. doi: 10.3390/ijms24021265 (PMC9860682; doi:10.3390/ijms24021265)
Supplement: Supplementary file 1 [file ijms-24-01265-s001.zip › Supplementary Materials.pdf]

Figure S1: Nr homologous species distribution.

Figure S2: GO enrichment analysis of the assembled isoforms.

Figure S3: Venn diagram of CNCI, CPC, CPAT and Pfam prediction results.

Figure S4: Transcription factor type distribution.

Figure S5: DEGs common at different time points. (A) KEGG pathway enrichment at the 3 h vs. CK; (B) KEGG pathway enrichment at 6 h vs. the CK; (C) KEGG pathway enrichment at 9 h vs. the CK. A pathway's rich factor is the ratio of the DEG value to the background value. Q-values and DEG numbers are indicated by the color and size of the dots.

Figure S6: Relative gene expression levels of select genes. The data shown are the means of three biological replicates (n = 3), and the bar represents the standard deviation (SD) for each mean value.
