# Supplementary material for: Molecular Mechanism of Cold Tolerance of Centipedegrass Based on the Transcriptome
Source: Int J Mol Sci. 2023 Jan 9;24(2):1265. doi: 10.3390/ijms24021265 (PMC9860682; doi:10.3390/ijms24021265)
Supplement: Supplementary file 1 [file ijms-24-01265-s001.zip › Table S1.docx]

**Table S1** Output statistics of second generation transcriptome sequencing

| Samples | Read Number | Base Number | GC Content | %≥Q30 |
| --- | --- | --- | --- | --- |
| C01 | 24,381,771 | 7,290,105,292 | 54.88 | 94.65 |
| C02 | 24,496,632 | 7,339,598,850 | 48.28 | 94.26 |
| C03 | 21,501,144 | 6,433,897,610 | 56.31 | 94.28 |
| C04 | 24,270,944 | 7,270,977,030 | 49.46 | 94.27 |
| C05 | 22,859,424 | 6,845,648,812 | 52.11 | 94.56 |
| C06 | 23,431,137 | 7,018,257,088 | 47.07 | 93.99 |
| C07 | 24,860,305 | 7,447,817,292 | 46.56 | 94.02 |
| C08 | 22,794,142 | 6,828,349,896 | 47.17 | 94.00 |
| C09 | 22,910,575 | 6,862,223,872 | 45.33 | 94.49 |
| C10 | 23,822,714 | 7,135,624,684 | 52.91 | 94.04 |
| C11 | 23,933,102 | 7,165,139,172 | 53.72 | 94.67 |
| C12 | 22,982,072 | 6,882,149,932 | 55.56 | 94.74 |
