# Supplementary material for: Molecular Mechanism of Cold Tolerance of Centipedegrass Based on the Transcriptome
Source: Int J Mol Sci. 2023 Jan 9;24(2):1265. doi: 10.3390/ijms24021265 (PMC9860682; doi:10.3390/ijms24021265)
Supplement: Supplementary file 1 [file ijms-24-01265-s001.zip › Table S2.docx]

**Table S2** Annotation statistics of seven databases

| Anno_Database | Isoforms Number |
| --- | --- |
| COG | 54,308 |
| GO | 92,875 |
| KEGG | 51,565 |
| KOG | 78,125 |
| Pfam | 99,431 |
| Swissprot | 68,221 |
| eggNOG | 118,100 |
| NR | 120,710 |
| All | 121,738 |
