# Supplementary material for: Molecular Mechanism of Cold Tolerance of Centipedegrass Based on the Transcriptome
Source: Int J Mol Sci. 2023 Jan 9;24(2):1265. doi: 10.3390/ijms24021265 (PMC9860682; doi:10.3390/ijms24021265)
Supplement: Supplementary file 1 [file ijms-24-01265-s001.zip › Table S3.docx]

**Table S3.** Summary of KEGG pathway functional annotations under cold stress treatment

| pathway | ID code | CK vs 3h | | | |  | CK vs 6h | | | |  | CK vs 9h | | | |
| --- | --- | --- | --- | --- | --- | --- | --- | --- | --- | --- | --- | --- | --- | --- | --- |
|  |  | total number | up | down | *P* value |  | total number | up | down | *P* value |  | total number | up | down | *P* value |
|  |  |  |  |  |  |  |  |  |  |  |  |  |  |  |  |
| Arginine and proline metabolism | ko00330 | 5 | 5 | 0 | 0.02580765 |  | - | - | - | - |  | - | - | - | - |
| Circadian rhythm - plant | ko04712 | - | - | - | - |  | 7 | 4 | 3 | 0.003210388 |  | 6 | 5 | 1 | 0.042089503 |
| Flavonoid biosynthesis | ko00941 | - | - | - | - |  | - | - | - | - |  | 5 | 4 | 1 | 0.001955402 |
| Plant hormone signal transduction | ko04075 | - | - | - | - |  | - | - | - | - |  | 11 | 9 | 2 | 0.002224382 |
| Total |  | 5 | 5 |  |  |  | 7 | 4 | 3 |  |  | 22 | 18 | 4 |  |
