# Supplementary material for: Molecular Mechanism of Cold Tolerance of Centipedegrass Based on the Transcriptome
Source: Int J Mol Sci. 2023 Jan 9;24(2):1265. doi: 10.3390/ijms24021265 (PMC9860682; doi:10.3390/ijms24021265)
Supplement: Supplementary file 1 [file ijms-24-01265-s001.zip › Table S4.docx]

**Table S4.** Primers used for RT-qPCR verification

| gene ID | Annotation | Primers (5'to3') |
| --- | --- | --- |
| U6 |  | F: CTCGCTTCGGCAGCACATATACT |
|  |  | R: ACGCTTCACGAATTTGCGTGTC |
| D_transcript_4325 | COR1 | F: ATGCTGGTGGCTATTCTG |
|  |  | R: GGTGGTGTACCTCCATAAG |
| D_transcript_83829 | COR410 | F: TATCACCGTGTGCTTCAA |
|  |  | TCCTACACAACATCCAGAC |
| D_transcript_71144 | COR410 | F: AAGTCAAGGTCCGTATCA |
|  |  | R: ACTCCTACACAACATCCA |
| D_transcript_68926 | RAB15 | F: TTGACTAAACACGCACGAAT |
|  |  | R: GGCAATTTCTCCTTGATCTTCT |
| D_transcript_27429 | CS66 | F: GGACAAGTACTGGATCATCA |
|  |  | R: CGTCCTTCCTCATCTTGAT |
| D_transcript_21882 | C3H2 | F: CATATCTATCGCCCTGTGAA |
|  |  | R: GATAATGTTCTCCGCTACCT |
| D_transcript_48115 | senescence-associated protein | F: GGCAGTAACAGCAGGTAG |
|  |  | R: TGGGACTGGAACATCTCT |
| D_transcript_23371 | senescence-associated protein | F: ATCCTGTCTGGTGTTCTGAAG |
|  |  | R: TCCATCAAGCGAAGCAAGA |
