# Supplementary figures and images for: Molecular Mechanism of Cold Tolerance of Centipedegrass Based on the Transcriptome
Source: Int J Mol Sci. 2023 Jan 9;24(2):1265. doi: 10.3390/ijms24021265 (PMC9860682; doi:10.3390/ijms24021265)

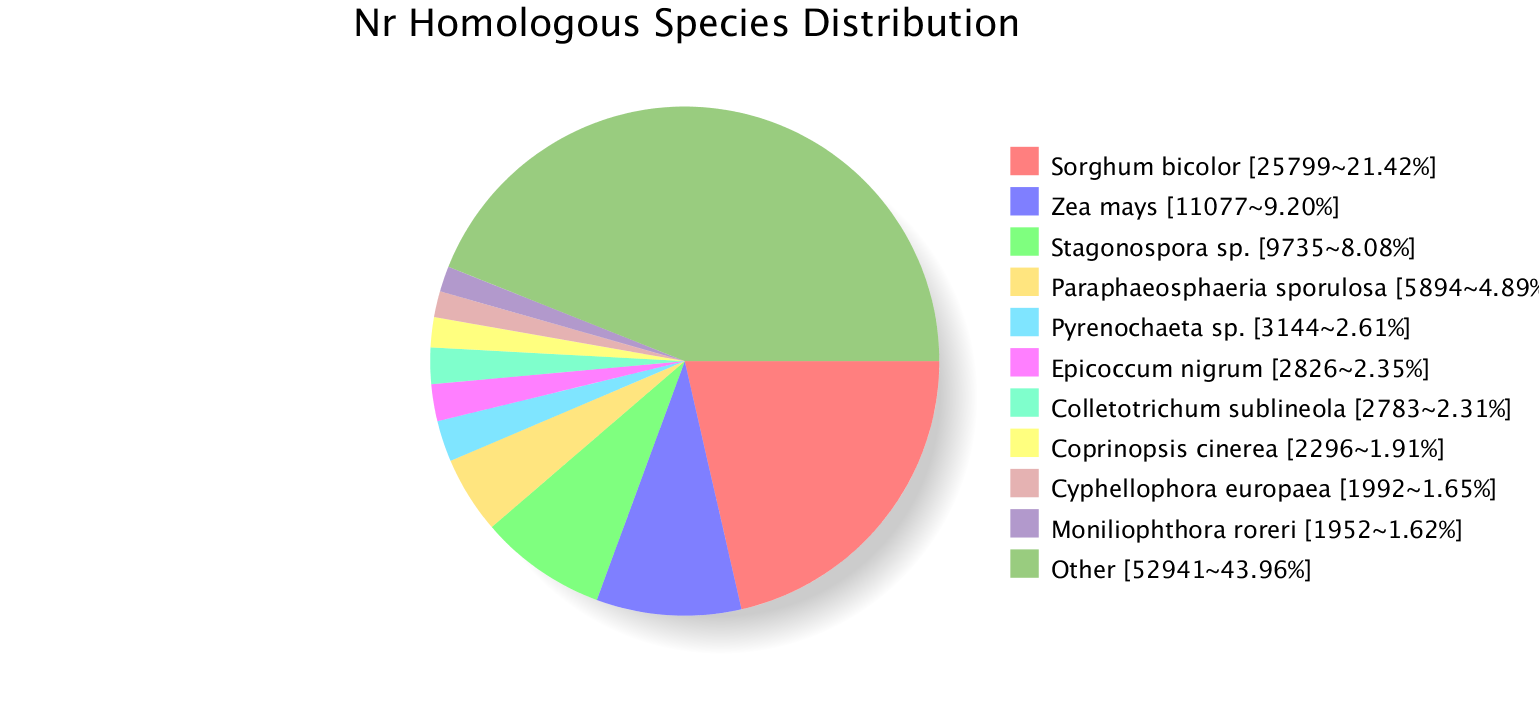

Supplement: Supplementary file 1 [file ijms-24-01265-s001.zip › Figure S1.tif]

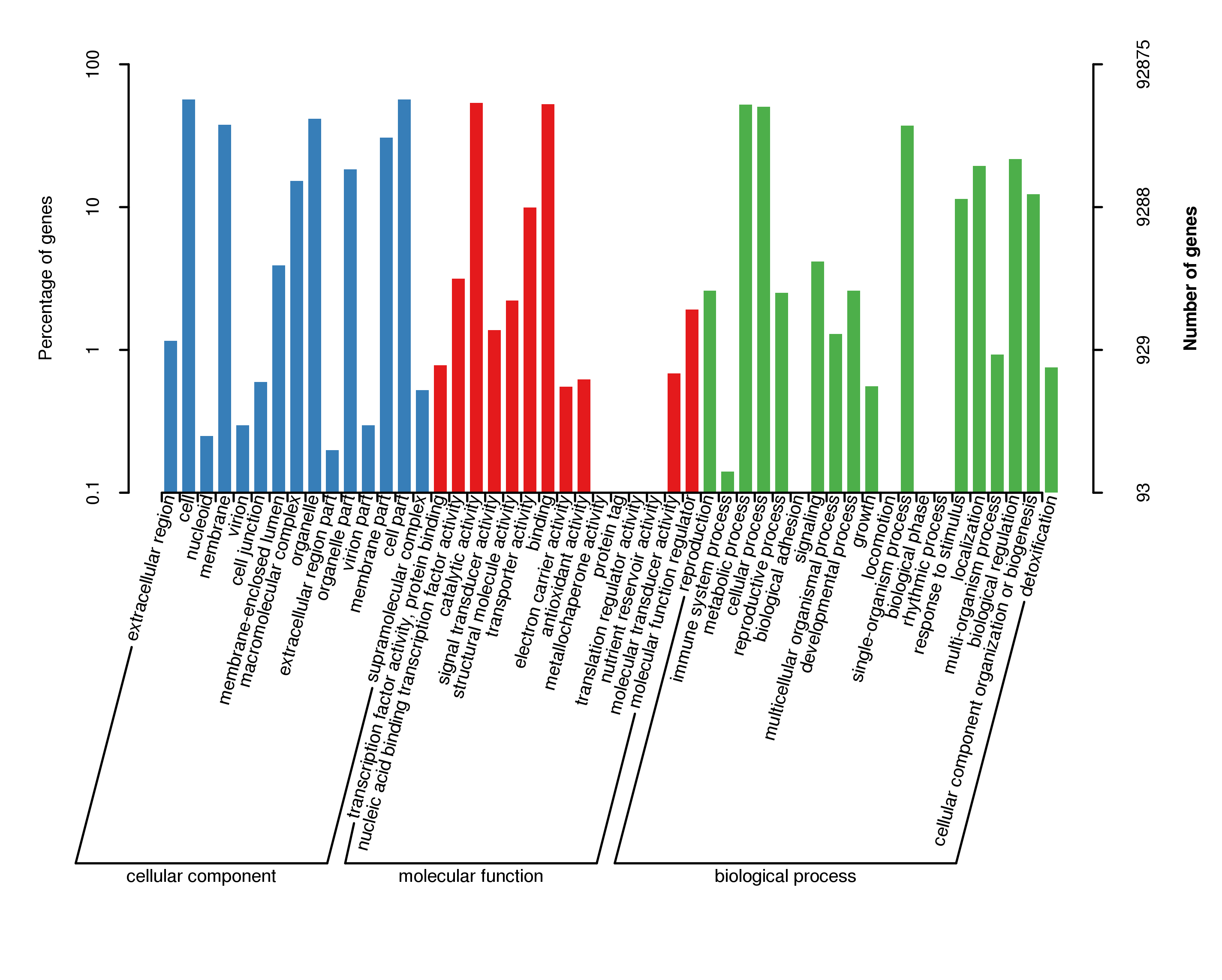

Supplement: Supplementary file 1 [file ijms-24-01265-s001.zip › Figure S2.tif]

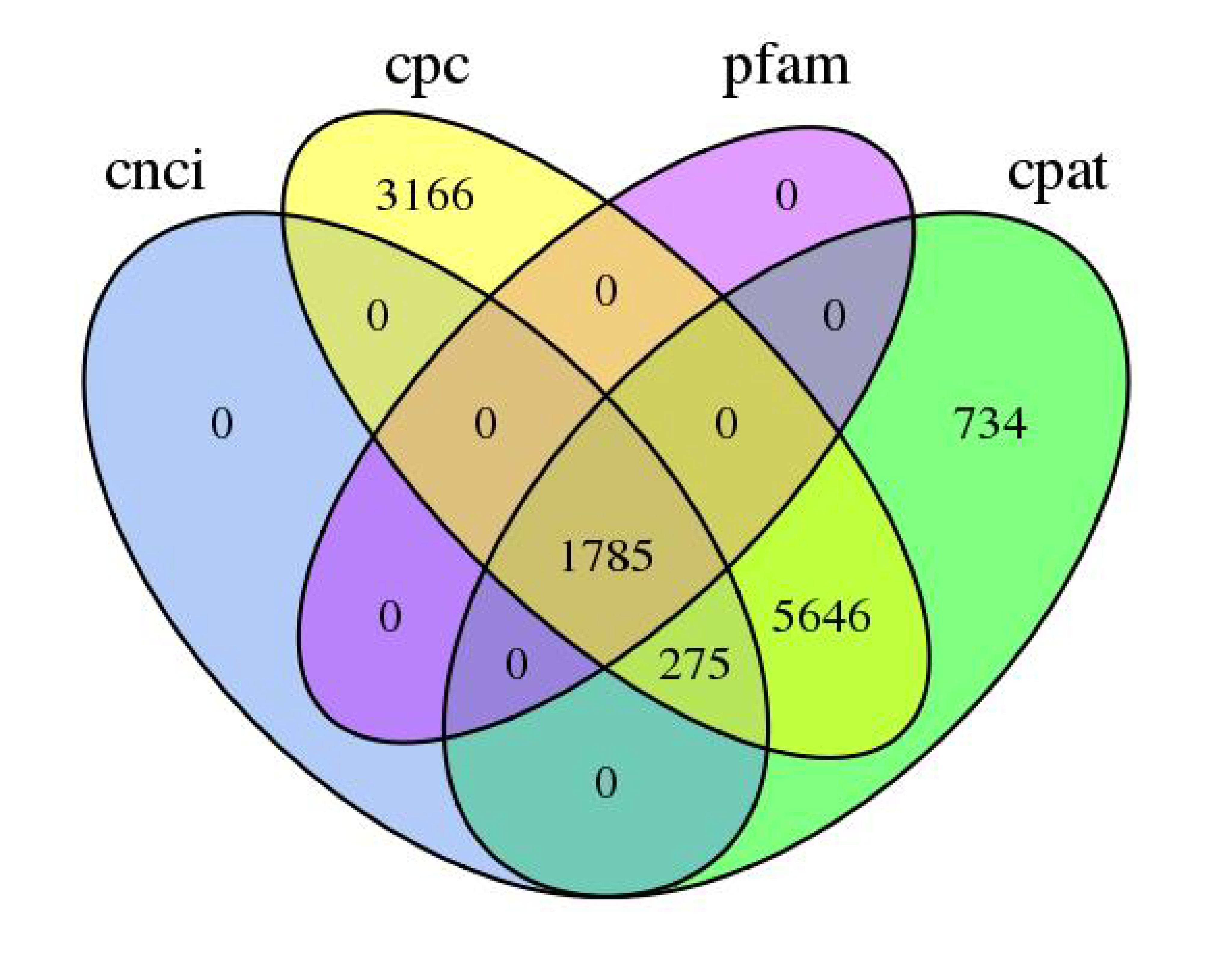

Supplement: Supplementary file 1 [file ijms-24-01265-s001.zip › Figure S3.tif]

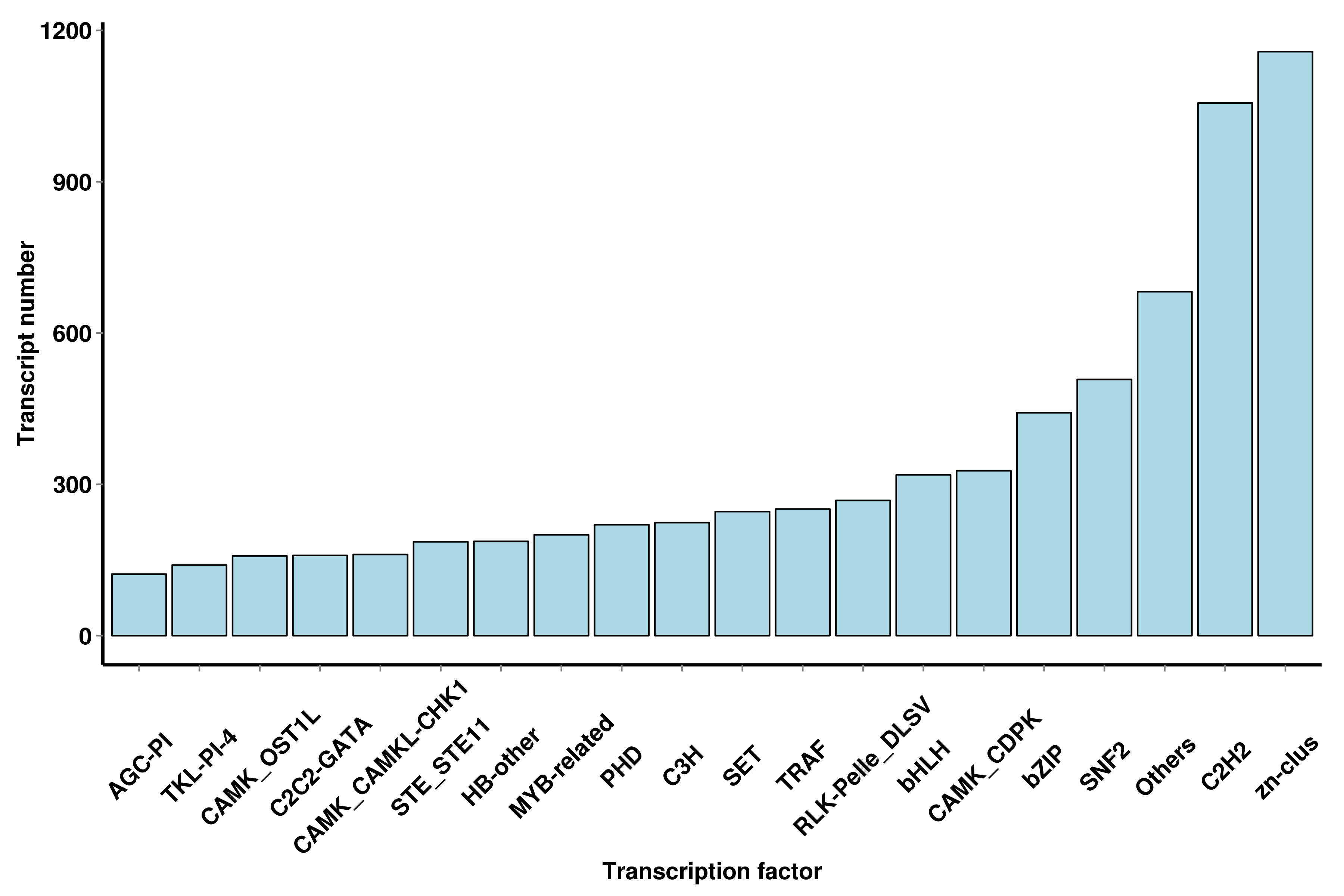

Supplement: Supplementary file 1 [file ijms-24-01265-s001.zip › Figure S4.tif]

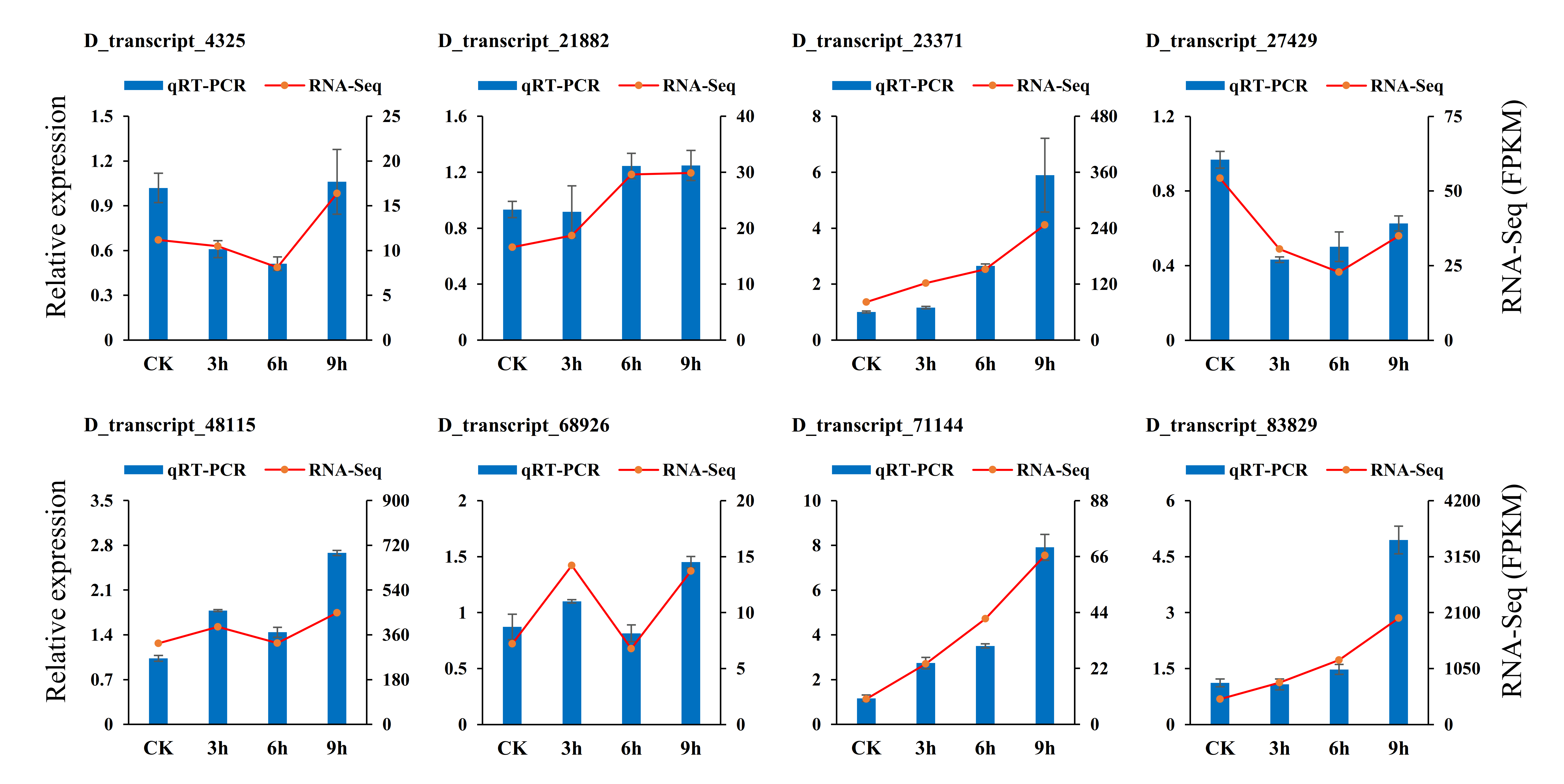

Supplement: Supplementary file 1 [file ijms-24-01265-s001.zip › Figure S6.tif]
